# Leveraging transcriptomics to develop bronchopulmonary dysplasia endotypes: a concept paper

**DOI:** 10.1186/s12931-023-02596-y

**Published:** 2023-11-15

**Authors:** Alvaro G. Moreira, Tanima Arora, Shreyas Arya, Caitlyn Winter, Charles T. Valadie, Przemko Kwinta

**Affiliations:** 1 Department of Pediatrics, Division of Neonatology, University of Texas Health San Antonio, San Antonio, TX USA; 2 Division of Neonatology, Dayton Children’s Hospital, Cincinnati, OH USA; 3 Neonatal Intensive Care Unit, Department of Pediatrics, Jagiellonian University Medical College, Kraków, Poland

## Abstract

**Impact:**

Bronchopulmonary dysplasia has multiple definitions that are currently based on phenotypic characteristics. Using an unsupervised machine learning approach, we created BPD subclasses (e.g., endotypes) by clustering whole microarray data. T helper 17 cell differentiation was the most significant pathway differentiating the BPD endotypes.

**Introduction:**

Bronchopulmonary dysplasia (BPD) is the most common complication of extreme prematurity. Discovery of BPD endotypes in an unbiased format, derived from the peripheral blood transcriptome, may uncover patterns underpinning this complex lung disease.

**Methods:**

An unsupervised agglomerative hierarchical clustering approach applied to genome-wide expression of profiling from 62 children at day of life five was used to identify BPD endotypes. To identify which genes were differentially expressed across the BPD endotypes, we formulated a linear model based on least-squares minimization with empirical Bayes statistics.

**Results:**

Four BPD endotypes (A, B,C,D) were identified using 7,319 differentially expressed genes. Across BPD endotypes, 5,850 genes had a p value < 0.05 after multiple comparison testing. Endotype A consisted of neonates with a higher gestational age and birthweight. Endotypes B-D included neonates between 25 and 26 weeks and a birthweight range of 640 to 940 g. Endotype D appeared to have a protective role against BPD compared to Endotypes B and C (36% vs. 62% vs. 60%, respectively). The most significant pathway focused on T helper 17 cell differentiation.

**Conclusion:**

Bioinformatic analyses can help identify BPD endotypes that associate with clinical definitions of BPD.

**Supplementary Information:**

The online version contains supplementary material available at 10.1186/s12931-023-02596-y.

## Introduction

Ever since its description over half a century ago, bronchopulmonary dysplasia (BPD) remains the most common complication associated with extreme prematurity [1]. Currently, BPD affects approximately 18,000 premature newborns per year and costs the United States $2.5 billion annually [2]. This chronic lung disease is characterized by an arrest in lung development secondary to chronic exposure to positive pressure ventilation and supplemental oxygen [3]. Histologically, BPD is hallmarked by a decreased number of lung alveolar cells, an immature capillary network, and increased fibrin deposition in the basal membrane [4]. The incidence of BPD is inversely proportional to gestational age and is associated with significant morbidity and mortality [5]. Long-term sequelae from BPD include cognitive deficits, hearing and/or visual impairment, and decreased pulmonary function [6]. Despite significant advances in neonatal care, therapies for BPD are largely ineffective and in fact rates continue to increase due to the higher probability of survival of smaller premature neonates [2].

Due to the evolution of the disease, and the advent of newer respiratory modalities, the definition of BPD has undergone several different iterations [7]. Broadly, BPD is defined by the need for supplemental oxygen at 28 days postnatal age or 36 weeks postmenstrual age and its severity is classified based on the mode of respiratory support used [8]. However, the shortcoming with all previous and current definitions of BPD is that they are largely clinical and pay no heed to the complex pathophysiological pathways at play in the development of different disease phenotypes. This highlights the need to better classify BPD to potentially improve: (i) the prediction of pulmonary outcomes in neonates, and (ii) the development of therapies that target the appropriate patients.

A bioinformatic approach towards identifying ‘endotypes’ of BPD has not been previously conducted. An endotype is defined as a subclass of a disease that is characterized by its unique pathobiological mechanism [9]. Leveraging computer science, biology, genetics, statistics, and mathematics with clinical data and “-omic” technology may offer a more comprehensive assessment of pathways underpinning the heterogeneity in phenotypic presentations of BPD [10]. For instance, Wong et al. have distinguished three endotypes for pediatric sepsis using genome-wide expressing profiling [11]. Through unsupervised hierarchical clustering of gene expression, they characterized a pediatric sepsis endotype that was associated with a higher illness severity and mortality rate. Gene pathways that were altered in this endotype included the adaptive immune system and glucocorticoid receptor signaling, which may serve as future targets for drug discovery.

Similar to BPD, asthma is a complex inflammatory airway disease that clinically manifests heterogeneously. In a review article focused on asthma endotyping, Anderson argues that the current manner in which new therapies for asthma patients are tested is flawed [12]. Specifically, he states the inclusion criteria of randomized trials for asthma patients are based on characteristics that can be readily measured (e.g., eosinophilia, forced expiratory volume in 1 s, IgE levels), instead of selecting patients that are most likely to respond to the new agent. Consequently, such processes result in high drug failure rates and outcomes that may not be generalizable to other asthma patients. He proposes establishing subclasses of the disease to produce more precise definitions/variants of asthma and the establishment of biomarkers and/or pathways that more accurately explain the intricacies of asthma.

Using publicly-available whole microarray data [13], we performed an unsupervised hierarchical clustering technique to identify BPD endotypes. Furthermore, we analyzed pathways that were unique to each endotype and examined their association to BPD severity. Finally, we conducted a supervised machine learning approach to determine an early discriminatory ability of BPD endotype employing the top differentially expressed genes.

## Methods

### Subjects

A secondary analysis of a microarray dataset (GSE32472) from the National Library of Medicine’s Gene Expression Omnibus (https://www.ncbi.nlm.nih.gov/geo/) was performed. The study was conducted in Poland between the years 2008 and 2010 and included preterm newborns with birthweight ≤ 1500 g and who required respiratory support at the time of enrollment. This dataset included 97 neonates. Further details regarding the dataset have been previously reported [13]. Peripheral blood sampling for microarray gene expression was examined on day of life five. Institutional review board approval was not required as this study used publicly available de-identified information. The primary objective was to create endotypes for bronchopulmonary dysplasia (BPD) using whole blood microarray gene expression data.

### Data analysis

#### Gene expression data preparation and analysis

Our first goal was to identify differentially expressed genes between neonates with or without BPD. Boxplots and histograms were created to assess for normal distribution of gene expression. We began from a working list of 33,252 genes per patient. Next, gene counts were log_2_ transformed followed by quantile normalization. Genes with expression levels < 50% of total expression from all samples were excluded as low levels across all samples are not likely to be differentially expressed. Outliers were weighted per Ritchie et al. [14]. Genes were considered significant if the false discovery rates (FDR), by Benjamini and Hochberg-adjusted P values, were less than 5%.

#### Unsupervised machine learning (ML) model

We utilized an unsupervised agglomerative hierarchical clustering approach to identify BPD endotypes. ‘Unsupervised learning uses algorithms to discover hidden patterns or data groupings without the need for human intervention’ [15]. Hierarchical clustering groups similar data in a continuous fashion until a difference is seen, at which point one cluster is formed and the beginning of another cluster ensues. Using the *cluster* package (version 2.2.2) in R, we created a dendrogram of the subclasses via Ward’s linkage using Euclidean distances. A priori, we decided to segment the clusters based on no more than third-order branching of the dendrogram. We chose Ward’s linkage as our clustering method as it is an established method for producing well-defined and compact clusters, which is particularly advantageous when identifying subtypes or endotypes within complex biological data [16]. Euclidean distance was chosen because it is a widely accepted metric that measures similarity or dissimilarity between data points [16].

Moreover, we utilized the *cluster* package in R as it offers a comprehensive suite of clustering methods and extensive visualization tools, making it a widely accepted and trusted tool in bioinformatics and data analysis [17]. To further visualize the BPD endotypes we conducted principal component analyses. Principal component analysis is a visual representation of a mathematical computation that decreases the dimension of data by like samples.

#### Differential gene expression across BPD endotypes

To identify which genes were differentially expressed across the BPD endotypes we formulated a linear model based on least-squares minimization with empirical Bayes statistics using the *limma* package in R statistical software version 4.1.0. The model calculated the log fold change, probability value, and adjusted probability values between the different endotype groups (Endotype A versus the average expression of Endotype B, C, and D; Endotype B versus average expression of Endotypes A, C, and D, etc.). This approach allowed for a systematic analysis to identify genes that show significant expression differences across the groups, providing insights into the molecular characteristics of each endotype. Volcano plots were plotted to graphically interpret the differences in gene expression between the endotypes. Phenotypic information was merged with genes with an adjusted p value < 1% to describe clinical information differing the BPD endotypes. BPD was divided into mild BPD vs. moderate/severe BPD. We defined BPD in this manner as the two most common BPD definitions use the timepoints at 28 days or 36 weeks. BPD at 28 days is captured by neonates with mild disease, while BPD at 36 weeks includes neonates with moderate to severe BPD. Another reason for a binary definition of BPD is that a small portion of the neonates in this study developed moderate or severe BPD.

#### Pathway analysis

Genes with an adjusted p value < 1% were grouped into gene ontologic pathways using the R package *gprofiler2*. Pathways were organized according to WikiPathways based on their log p adjusted values. The program performs functional enrichment analysis based on the genes inputted. Afterwards, ShinyGo 0.76.3, an open-source software platform for visualizing complex networks, was used to create a map to demonstrate the interactions of the biologic pathways (http://bioinformatics.sdstate.edu/go/) [18].

#### Top genes to identify BPD endotypes

To reduce the number of differentially expressed genes that can be used to predict BPD endotypes, we performed supervised machine learning. Specifically, we carried out a random forest split using the *Boruta* package in R using differentially expressed genes (e.g., Endotype A versus average expression of other Endotypes B, C, and D; Endotype B versus average expression of Endotypes A, C, and D, etc.) with a q value < 1%. The random forest machine learning algorithm is ‘an ensemble learning algorithm, which is a combination of multiple base decision trees’ (page 1104) [19]. The top 20 genes, based on adjusted p values, were used in a multivariate adaptive regression spline (MARS) algorithm to examine their predictive performance in discriminating the BPD endotypes. We used MARS as it has an automatic feature selection for best predictors. The data was split into a training (2/3) and test cohort (1/3). To minimize overfitting, we used 10-fold cross-validation repeated five times. Default hyperparameters within the caret package in R was used and no data were imputed. Metrics used to test the predictive performance of the model included: sensitivity, specificity, positive predictive value, negative predictive values, and the area under the receiver operating characteristic curve. Finally, we used a heatmap and boxplot to picture the gene differences across the BPD endotypes.

## Results

### Genes differentially expressed in neonates with BPD

To identify BPD subclasses, we first had to identify genes that were differentially expressed in preterm infants with or without BPD. Of the 97 very low birthweight neonates, 62 (63.9%) were diagnosed with BPD. The whole microarray data included 33,252 genes per patient. Gene expression levels less than the median for all samples were removed. After multiple comparison testing, by adjusting p values via the Benjamini and Hochberg method, 7,319 genes (22%) had an FDR < 0.05 (Supplemental File 1).

### Development of BPD endotypes

**Fig. 1:**
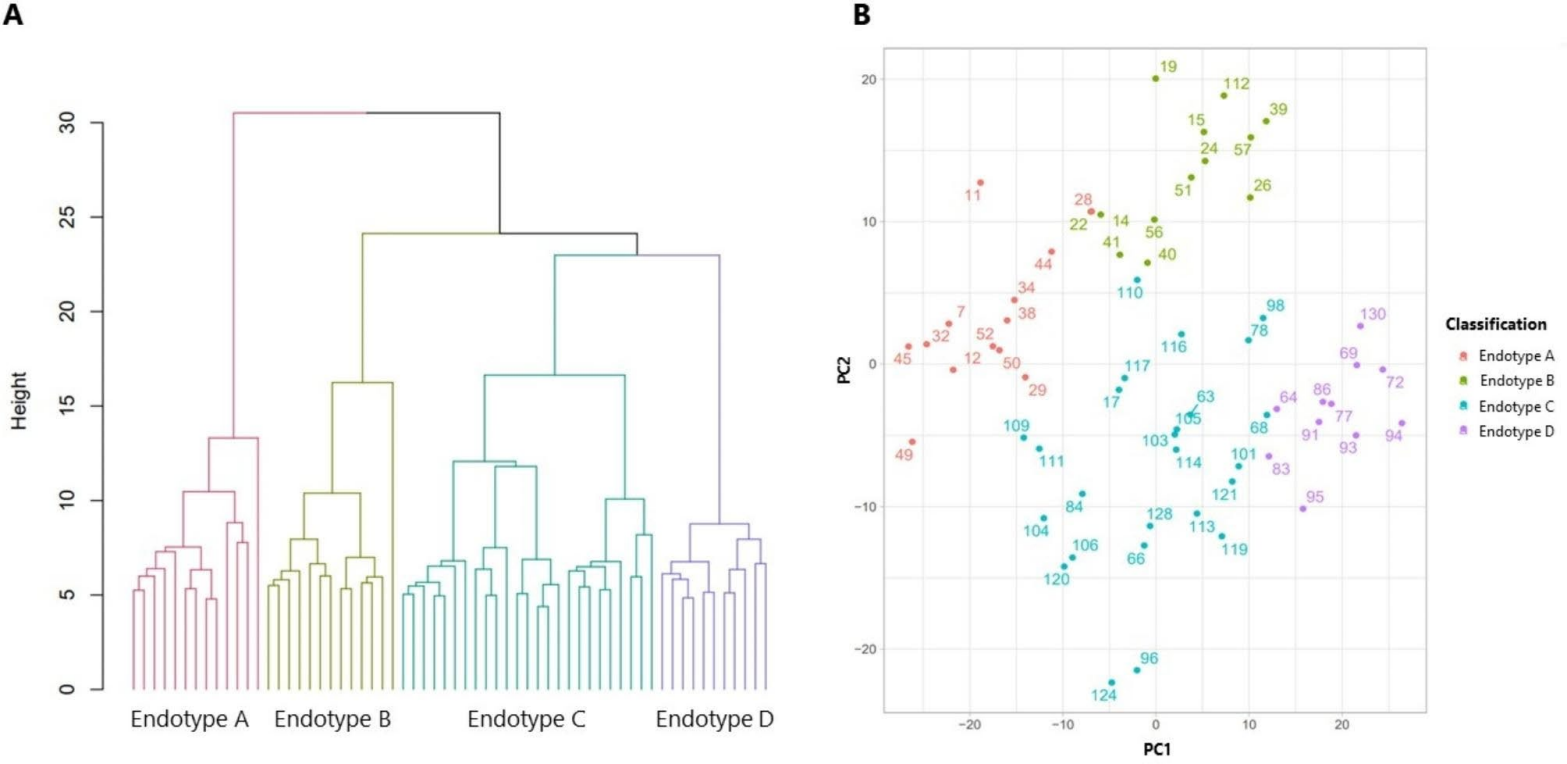
**A**) Unsupervised hierarchical clustering of 62 neonates with BPD demonstrated as a dendrogram. Agglomerative clustering with Euclidean distances and Ward’s linkage was used to create four endotypes up to the third-order branching patterns of the condition tree. **B**) Principal components analysis demonstrating spatial separation among the BPD endotypes based on 7,319 genes differentially expressed in BPD

Agglomerative unsupervised hierarchical clustering was performed on the 7,319 differentially expressed genes to classify the potential BPD endotypes. A priori we decided that no more than three branches from the dendrogram would be used to stratify the subclasses. Figure 1 depicts the four BPD endotypes, that were arbitrarily assigned the names A, B, C, and D, as a dendrogram and principal components analysis.

### Differentially expressed genes across BPD endotypes

**Fig. 2:**
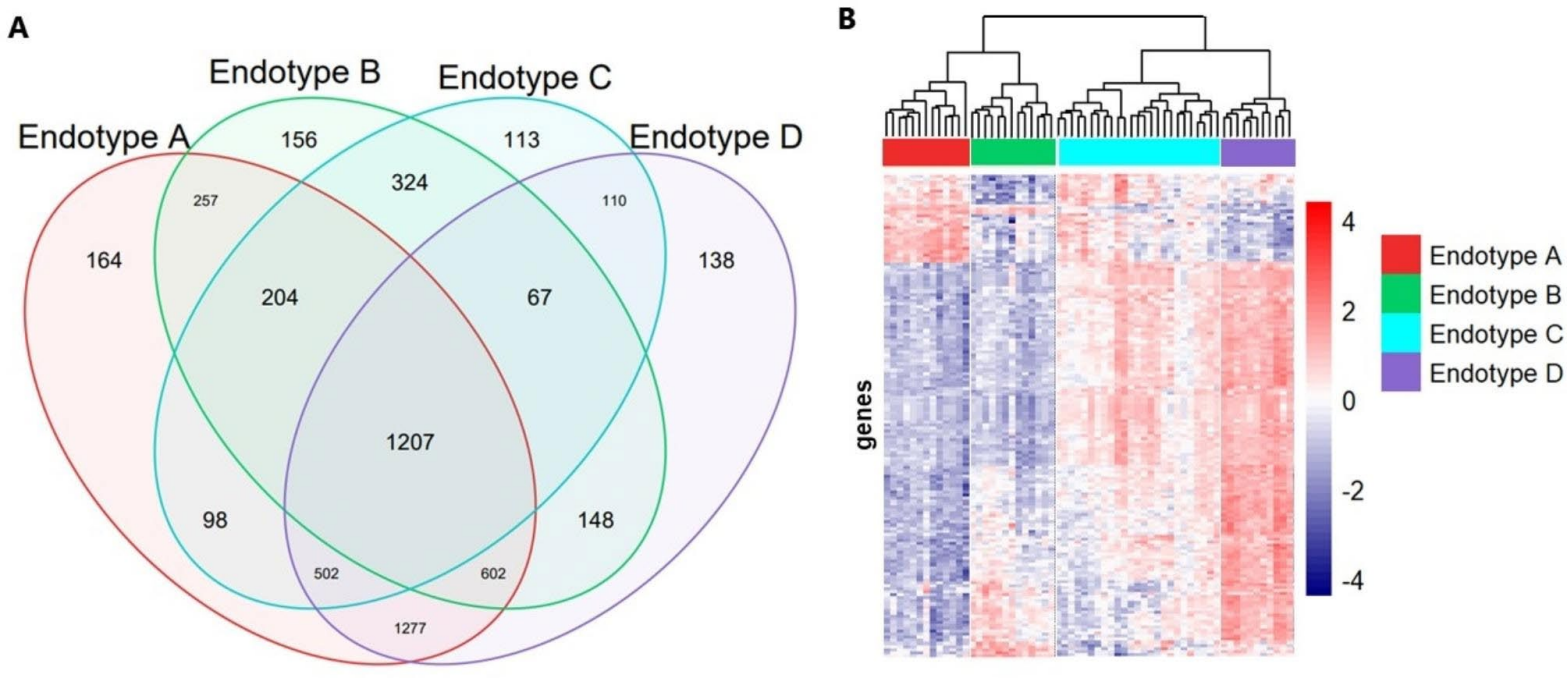
**A**) Venn diagram illustrating overlap/separation of genes by BPD endotype. **B**) Unsupervised hierarchical clustering of 62 neonates with BPD and heatmap summarizing peripheral blood gene expression on day 5 across endotypes. Each column represents a sample and the four endotypes are clustered according to color. Each row represents a gene and the colors indicate the magnitude of expression (all genes have been log_2_ transformed and quantile normalized). Blue denotes low gene expression and red represents high expression

Using linear models and empirical Bayes methods, genes were tested for significance across the four endotypes. Five thousand eight hundred fifty-eight genes had a p value < 5% after multiple comparison testing. Supplemental Fig. 2 shows volcano plots depicting the differentially expressed genes according to BPD endotype (e.g., Endotype A versus average expression of other Endotypes B, C, and D; Endotype B versus average expression of Endotypes A, C, and D, etc.). Fig. 2 depicts the differentially expressed genes across the BPD endotypes via Venn diagram and a heatmap. The number of genes that were differentially expressed in each cluster were 4,311, 2,965, 2,625, and 4,051 for Endotype A, Endotype B, Endotype C, and Endotype D, respectively. Whereas, 1,207 genes were differentially expressed in all endotypes. To work with a more manageable dataset we opted to include only genes with a p value < 1% (n = 4,553 genes).

### Phenotypic characteristics of BPD endotypes

The BPD case group consisted of 62 neonates. Table 1 provides the demographic characteristics of the cohort separated by the four endotypes. Endotype A was comprised of neonates with a larger gestational age and birthweight. As expected, they had the lowest rates of moderate/severe BPD (7.7%). Endotypes B, C, and D had gestational ages between 25 weeks to 26 weeks and a birthweight range between 690 and 940 g. Interestingly, Endotype D had a low rate of moderate/severe BPD compared to Endotypes B and C (36% vs. 62% vs. 60%, respectively).

**Table 1:** Patient characteristics according to proposed BPD endotypes

Variable	Endotype A (n = 13)	Endotype B (n = 13)	Endotype C (n = 25)	Endotype D (n = 11)	P value
Gestational age (weeks)	28.0 (26.0, 29.0)	25.0 (24.0, 26.0)	26.0 (25.0, 29.0)	26.0 (25.0, 26.0)	0.017
Birthweight (grams)	950 (800, 1,140)	690 (600, 800)	940 (720, 1,030)	800 (700, 850)	0.017
Sex					0.3
Girl	7 (54%)	4 (31%)	7 (28%)	6 (55%)	
Boy	6 (46%)	9 (69%)	18 (72%)	5 (45%)	
BPD Severity					0.007
Mild	12 (92%)	5 (38%)	10 (40%)	7 (64%)	
Moderate/severe	1 (7.7%)	8 (62%)	15 (60%)	4 (36%)	

Continuous variables described as median (IQR); Categorical variables described as n (%)

Statistics used included Kruskal-Wallis rank sum test and Fisher’s exact test

### Pathways involved in differentially expressed genes across BPD endotypes

**Fig. 3:**
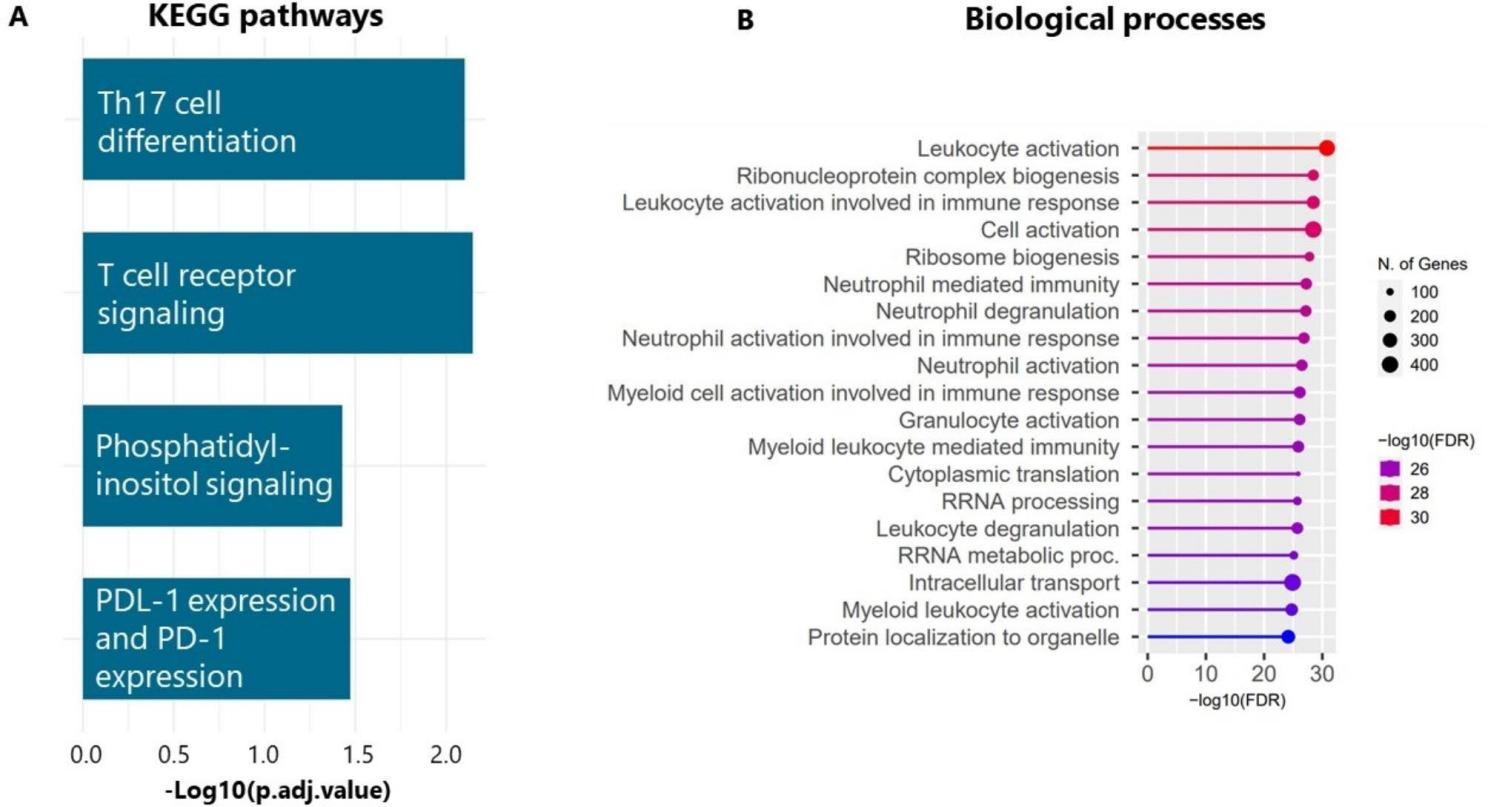
**A**) Barplot describing gene ontologic processes involved in the 4,553 differentially expressed genes (q value < 1%) expressed between the BPD endotypes by the negative log false discovery rate. **B**) Biologic processes involved in the differentially expressed genes within the BPD endotypes

The 4,553 differentially expressed genes represented part of four Kyoto Encyclopedia of Genes and Genomes (KEGG) pathways seen in Fig. 3A. The pathway with the largest log adjusted value dealt with T helper 17 cell differentiation, followed by T cell receptor signaling. The genes aligning with the T helper 17 cell differentiation pathway can be viewed in Supplemental Fig. 3. Fig. 3B highlights the biological processes involved in the differentially expressed genes. Eleven of the 19 (57.9%) processes focused on the immune system. Specifically, neutrophil activation or degranulation were among the top processes.

### Predicting endotype on day of life 5

**Fig. 4:**
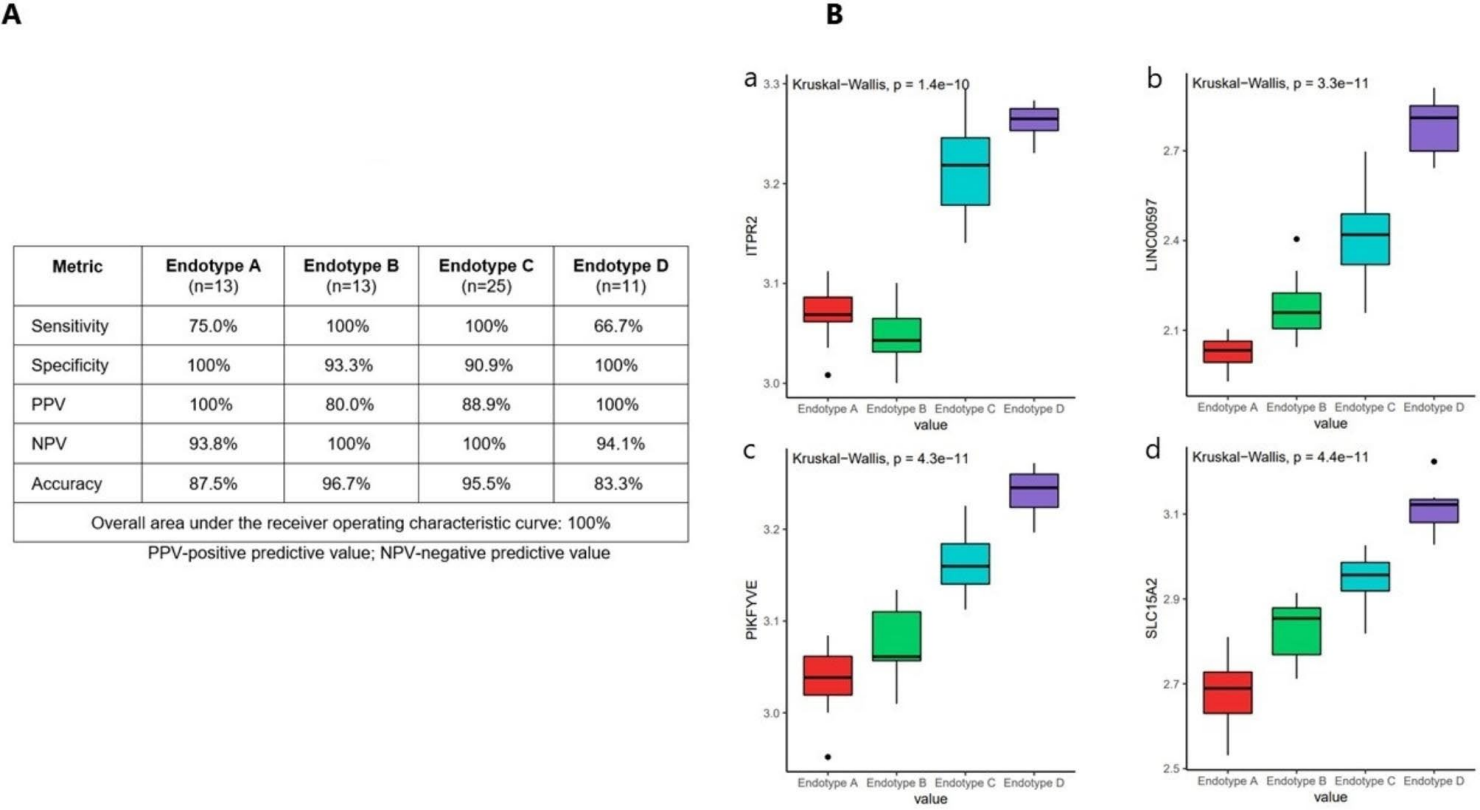
**A**) Performance metrics of top 20 genes used to predict BPD endotype on day 5. **B**) Boxplots with median and interquartile range of expression of four genes identified via machine learning that discriminates the BPD endotypes. Kruskal-Wallis test used to assess statistical differences among groups

Using supervised machine learning, we identified 20 genes that could serve as predictors for BPD endotype on day of life 5 (Fig. 4A). The table summarizes the discriminatory ability of the genes. To decrease the number of genes used to separate the endotypes, we passed a MARS algorithm on the top 20 genes (Supplemental Figs. 4 and 5). Fig. 4B illustrates the genes that were automatically selected as the best predictors for stratifying BPD endotypes. Overall, Endotype A had the lowest normalized expression of these genes, while the subsequent endotypes had a gradual increase in gene expression.

## Discussion

We have identified four BPD endotypes using whole genome microarray data from peripheral blood obtained in the first week of life. Pathway analysis clarified that T helper cell and T cell signaling distinguishes the BPD endotypes. We then identified a simplified combination of four genes that may be used for targeted discrimination across the BPD endotypes. Overall, these findings suggest that peripheral blood-based transcriptomics, combined with machine learning methods may help identify BPD subclasses in premature neonates.

Despite over 50 years of studying BPD, effective therapies for this condition are largely lacking [20]. Data derived from preclinical work and small-sized pilot studies have translated to multiple clinical trials; however, most of these studies have failed to show a reduction in BPD rates [21]. Reasons for these failures include studies targeting neonates with set inclusion criteria that focus on phenotypic information that does not necessarily correlate with the underlying diseases processes [22]. For example, most of the studies will include neonates with a birthweight ≤ 1500 g or a gestational age less than 32 weeks. However, our work suggests that more targeted efforts to identify those neonates most likely to benefit from particular interventions are needed, and that these efforts should focus on characteristics that can be directly related to disease processes. Phenotypic classification of patient populations is not enough- as we demonstrate here, transcriptomic data should be leveraged to produce a holistic understanding of patient population structure and generate appropriate inclusion or exclusion criteria.

In this case, although Endotypes B, C, and D, had similar gestational ages, two of the groups (B and C) had a much higher rate of moderate-severe BPD. These are the neonates that should be targeted in clinical trials. Early, novel intervention in these specific populations may demonstrate BPD mitigation, while continuing with standard of care for neonates belonging to Endotypes A and D could help save valuable resources when designing future clinical trials. Identification of BPD subclasses could thus aid in developing therapies that are more precise because the endotypes are surrogates of the underlying mechanisms of a particular neonate’s lung disease [22].

Long known to be critical influencers of lung development, the T-cell receptor signaling pathways are complex networks of molecular interactions responsible for maintaining the balance between innate and adaptive immunity [23]. In preterm infants, the immature immune system must contend with a sudden barrage of environmental pathogens and invasive medical interventions in a setting of hemodynamic instability, metabolic dysfunction, and oxidative stress [24]. Additionally, because prenatal inflammatory insults often contribute to preterm birth, many preterm infants have already experienced an intrauterine immune challenge before they even encounter the extrauterine environment [25]. Varying combinations of these endogenous and exogenous inflammatory risk factors interact with the newborn biome to produce the variety of disease phenotypes that are observed in BPD [20].

Within our cohort, endotypes A and D both had reduced incidence of severe disease, but as seen on the heatmap in Fig. 2B, patterns of gene expression in these endotypes appear to mirror each other, with endotype A exhibiting reduced expression where endotype D exhibits increased expression, and vice versa. While we must consider the role of gestational age and increased lung maturity in endotype A, it appears that an attenuated early inflammatory response may represent the most beneficial strategy for prevention of severe BPD. This is consistent with many studies which have shown that increases in T helper 2 induced cytokines are associated with BPD [13, 25–27]. However, among the infants born more prematurely (endotypes B, C, and D) it appears that an early, robust inflammatory response may be protective against development of severe BPD (endotype D). Indeed, Abalavanan et al. found that lower concentrations of interleukin-17 in the blood were associated with BPD or death [28]. Moreover, this large study also found that an impaired transition from the innate immune response via neutrophil activation associated with BPD or death. Similarly, when we assessed biologic processes altered on day of life 5 we found that neutrophils were critical in the protection/development of BPD. Because these data represent one time point only, we cannot say with certainty, but it seems likely that the inflammatory response in group D must be transient, else we would expect to see increased lung injury and arrested development in the setting of an uncontrolled inflammatory response. Humberg et al. explain the effects of such “sustained inflammation” as a moderator between survival and long-term morbidities in preterm infants [29].

To some extent, endotypes B and C also appear to have mirrored expression patterns, although the effect is less dramatic, the magnitude of differences in gene expression levels in these endotypes appears to be smaller. Still, these endotypes are associated with the highest rates of severe disease despite the fact that they contain infants of similar sizes and gestational ages. These endotypes do tend to have more males, although this difference was not statistically significant. Male sex has often been associated with poorer respiratory outcomes [2, 8, 26]. Endotype B exhibits an overall modest decrease in gene expression, which may represent a maladaptive anti-inflammatory response or immune exhaustion. In particular, endotype C appears to be quite mixed, with greater variation in all measured parameters. As this is also the most common endotype in our cohort, and has the second-highest rate of severe disease, future studies should focus on untangling this variation.

A major challenge in the implementation of precision medicine is the assessment of disease parameters for the identification of patients most likely to benefit from a particular treatment. Because it would be highly impractical and inefficient to perform transcriptomic profiling of all the genes for clinical diagnostics, we developed a simplified algorithm based on four genes that can discriminate between the four BPD endotypes. This algorithm would utilize a small peripheral blood sample, even a blood spot, to classify infants by BPD endotype as early as day of life five.

Although our work shows promise, our study does have limitations. For example, our study includes a small number of neonates with BPD from a homogeneous population derived from a single center. Validation of our model in an external cohort of neonates would strengthen the generalizability of our findings. Another limitation is that our data is retrospective in nature and would need to be reproduced in a prospective manner. Strengths of this study include leveraging bioinformatics with artificial intelligence to develop BPD endotypes for the first time. Furthermore, we used an unsupervised algorithm to identify patterns within the genes to decrease selection bias that often occurs when using a supervised approach [30]. We also generated a simplified signature of four genes that can potentially be used for early classification of infants into our BPD endotypes, with implications for individually-tailored intervention strategies.

In the future, it will be also important to understand how gene expression levels and associated biological pathways within the proposed endotypes may change over time, in order to identify targets for interventions. It will also be important to determine the relationships between BPD endotypes and clinical factors such as prenatal infection, exposure to corticosteroids, and postnatal medical interventions including ventilation strategies and nutritional support [29, 31]. A recent abstract by Ofman et al., employed a similar unsupervised machine learning algorithm for BPD endotyping. However, their analysis focuses on clinical data and not bioinformatic data [29]. BPD remains a complex and costly disease with long-term implications on an individual’s health and quality of life [30]. As medical technology continues to improve, allowing for the survival of increasingly smaller, sicker babies, the impact of BPD on global health will only expand. New therapeutic and preventive strategies are desperately needed to combat the detrimental effects of this disease. Emerging multi-omic technologies can provide the multifaced insight needed to meet these challenges.

### Electronic supplementary material

Below is the link to the electronic supplementary material.

**Additional file 1: Supplementary Fig.1.** Volcano plot of BPD vs. no BPD. Genes in teal color signify adjusted p value < 0.05

**Additional file 2: Supplementary Fig.2.** Volcano plot showing discrimination of genes across BPD endotypes

**Additional file 3: Supplementary Fig.3.** Th17 KEGG pathway with genes differentially expressed in dataset highlighted in red

**Additional file 4: Supplementary Fig.4.** Variable importance of multivariate adaptive regression spline model to discriminate between BPD endotypes

**Additional file 5: Supplementary Fig. 5**. Boxplots with median and interquartile range of expression of top 20 genes identified via machine learning that discriminates the BPD endotypes. Kruskal-Wallis test used to assess statistical differences among groups

## Data Availability

The datasets generated during and/or analyzed during the current study are publicly available on Gene Expression Omnibus (GEO) at https://www.ncbi.nlm.nih.gov/geo/ under the Accession: GSE32472.
