# Supplementary figures and images for: Leveraging transcriptomics to develop bronchopulmonary dysplasia endotypes: a concept paper
Source: Respir Res. 2023 Nov 15;24:284. doi: 10.1186/s12931-023-02596-y (PMC10648631; doi:10.1186/s12931-023-02596-y)

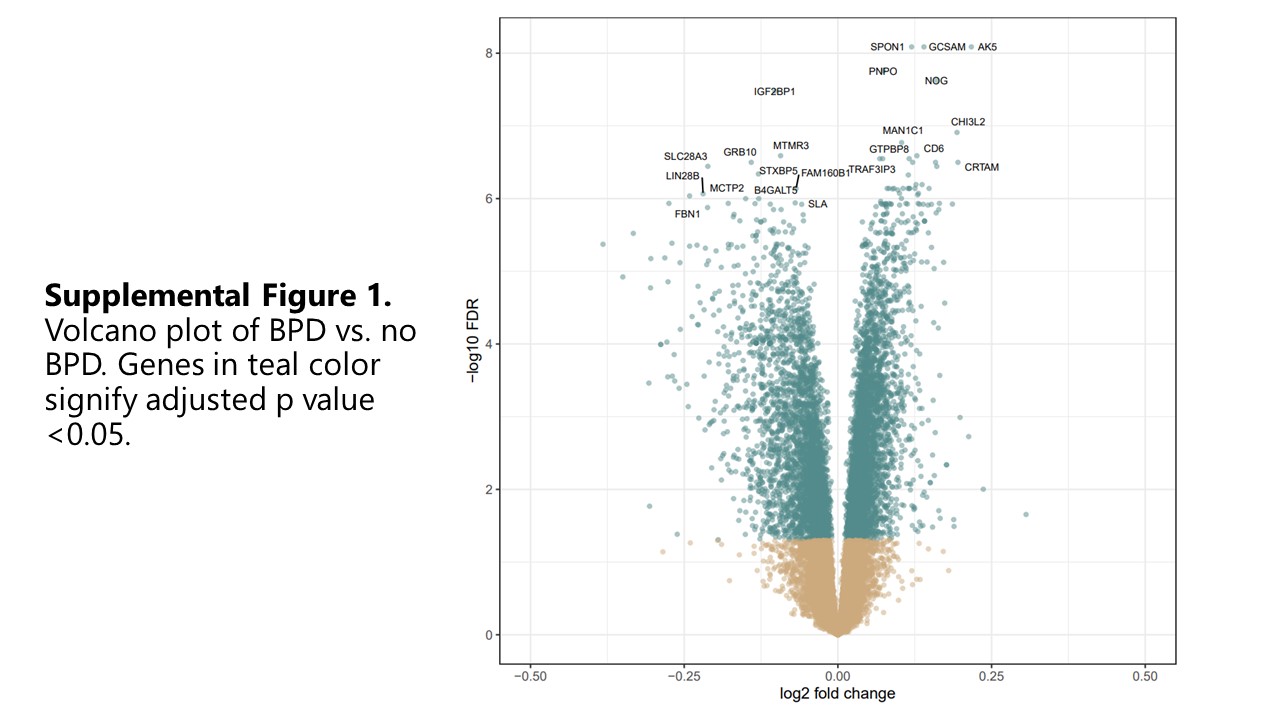

Supplement: Supplementary file 1 — Additional file 1: Supplementary Fig.1. Volcano plot of BPD vs. no BPD. Genes in teal color signify adjusted p value < 0.05 [file 12931_2023_2596_MOESM1_ESM.jpg]

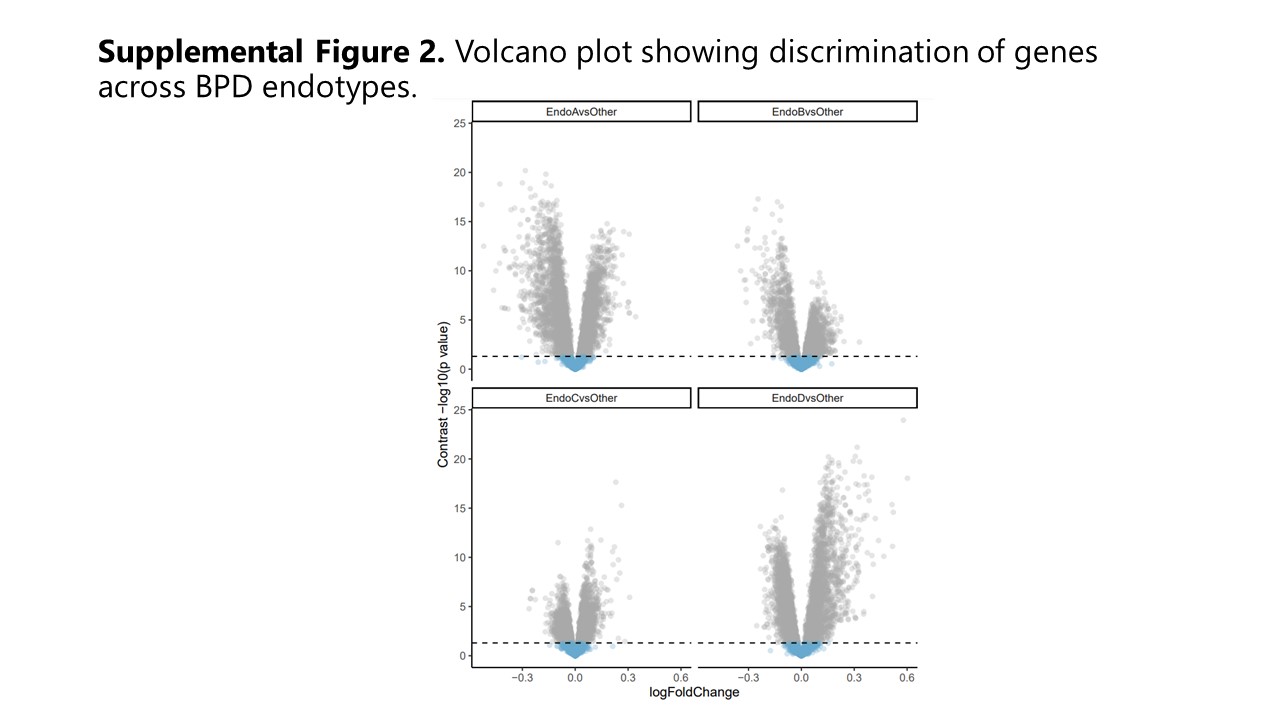

Supplement: Supplementary file 2 — Additional file 2: Supplementary Fig.2. Volcano plot showing discrimination of genes across BPD endotypes [file 12931_2023_2596_MOESM2_ESM.jpg]

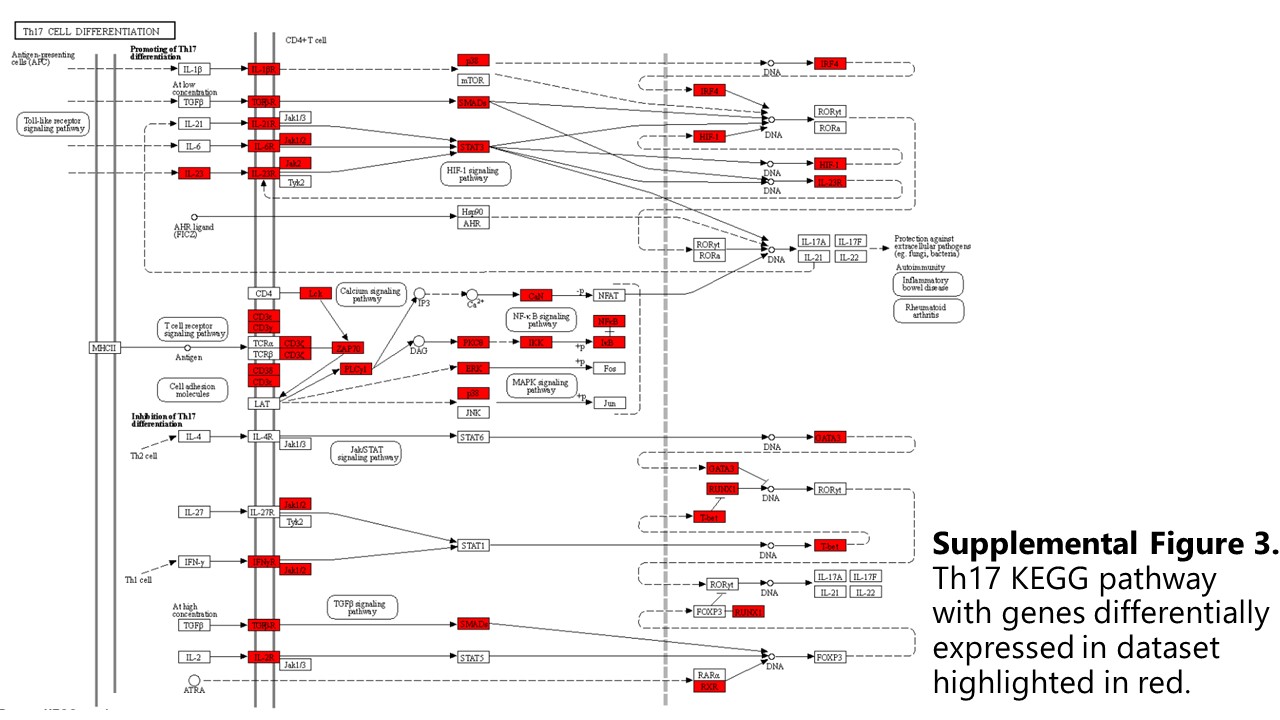

Supplement: Supplementary file 3 — Additional file 3: Supplementary Fig.3. Th17 KEGG pathway with genes differentially expressed in dataset highlighted in red [file 12931_2023_2596_MOESM3_ESM.jpg]

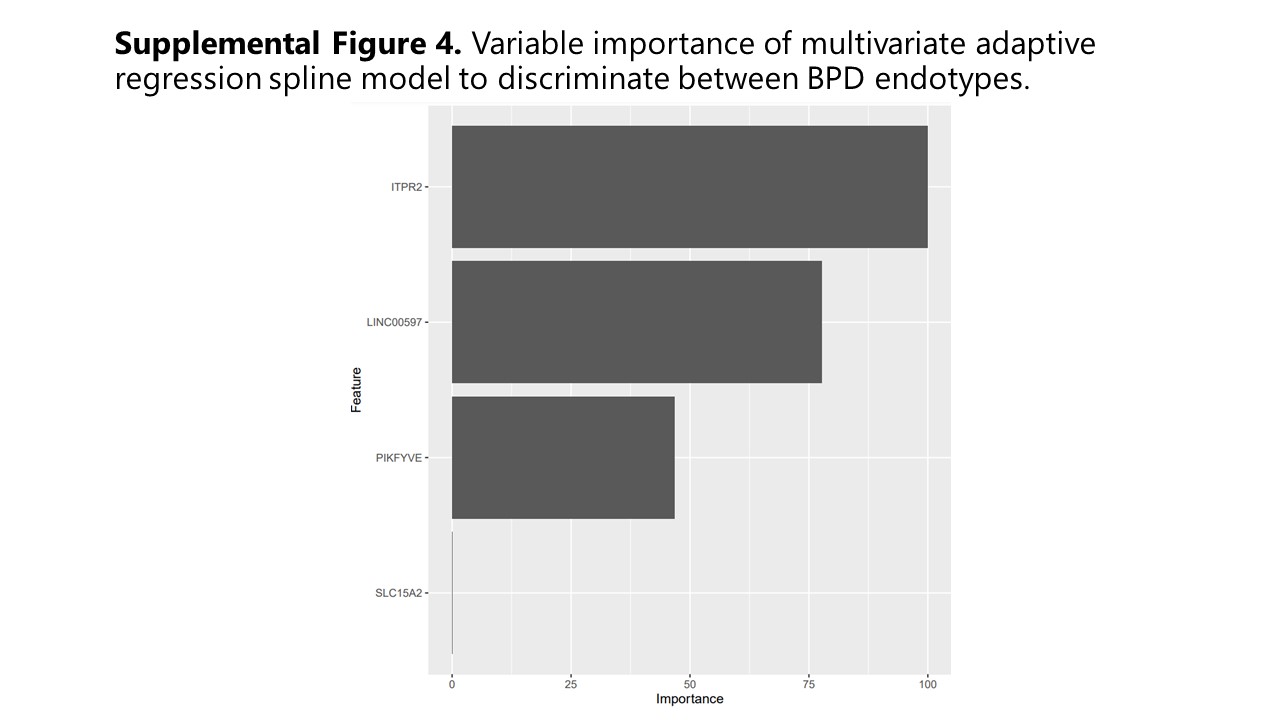

Supplement: Supplementary file 4 — Additional file 4: Supplementary Fig.4. Variable importance of multivariate adaptive regression spline model to discriminate between BPD endotypes [file 12931_2023_2596_MOESM4_ESM.jpg]

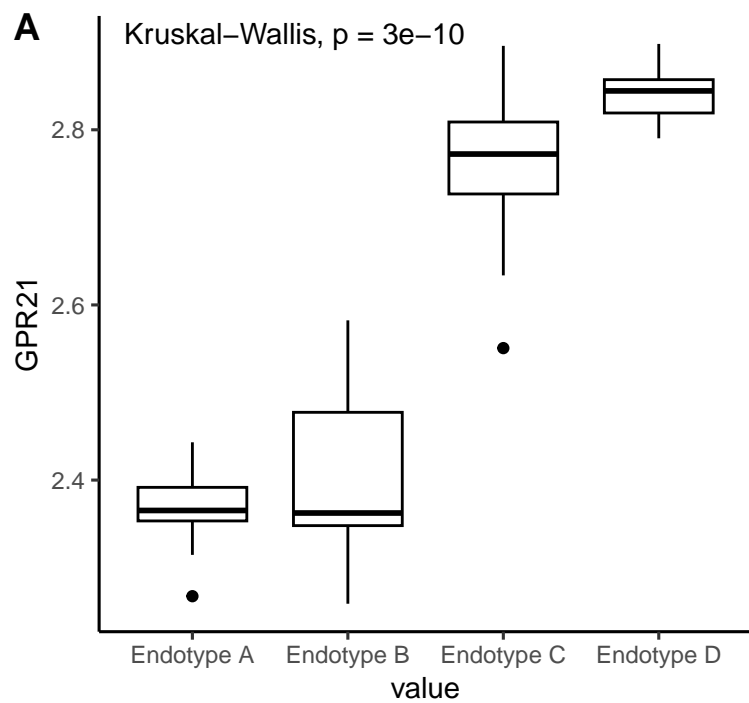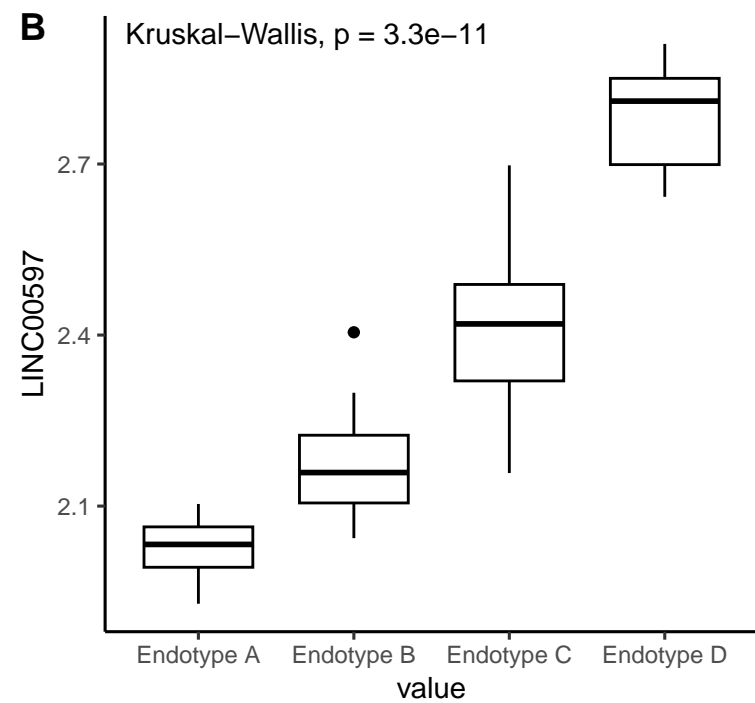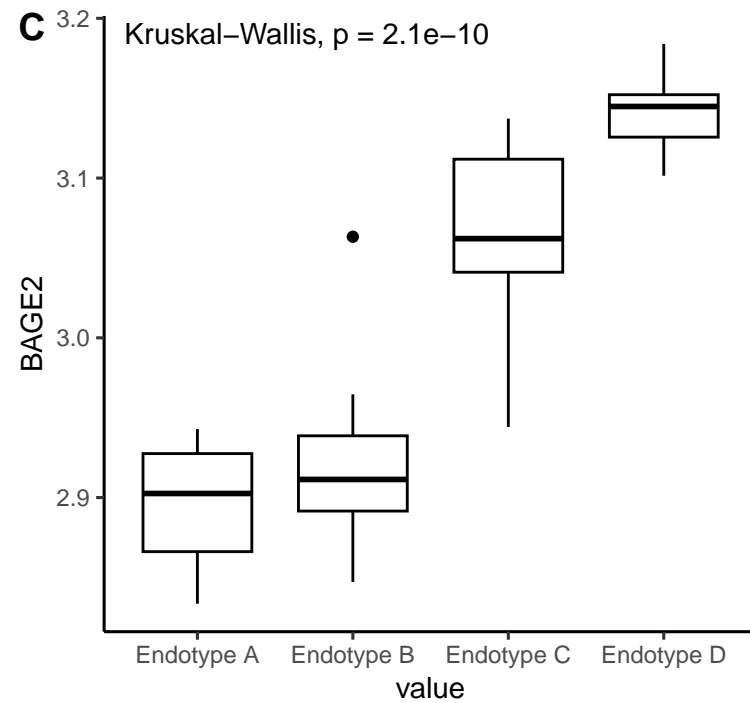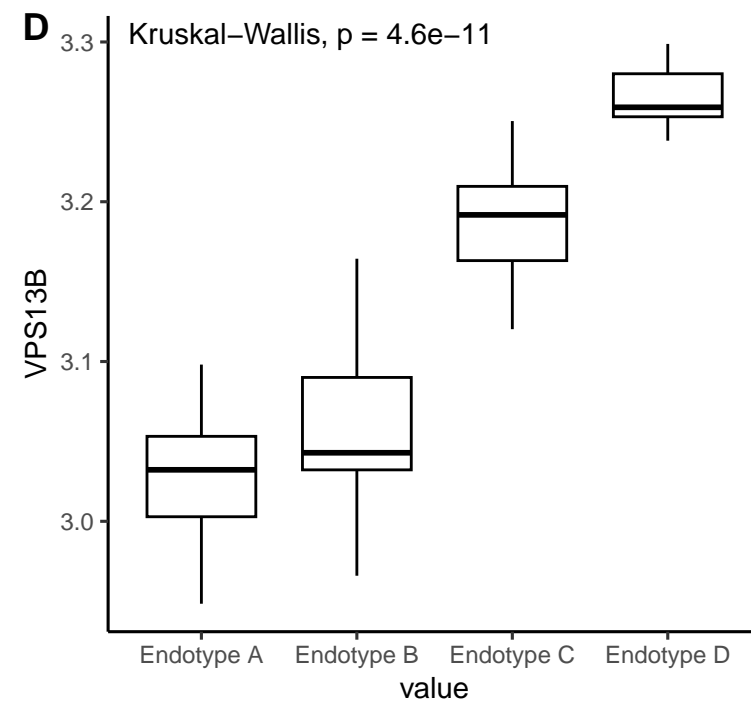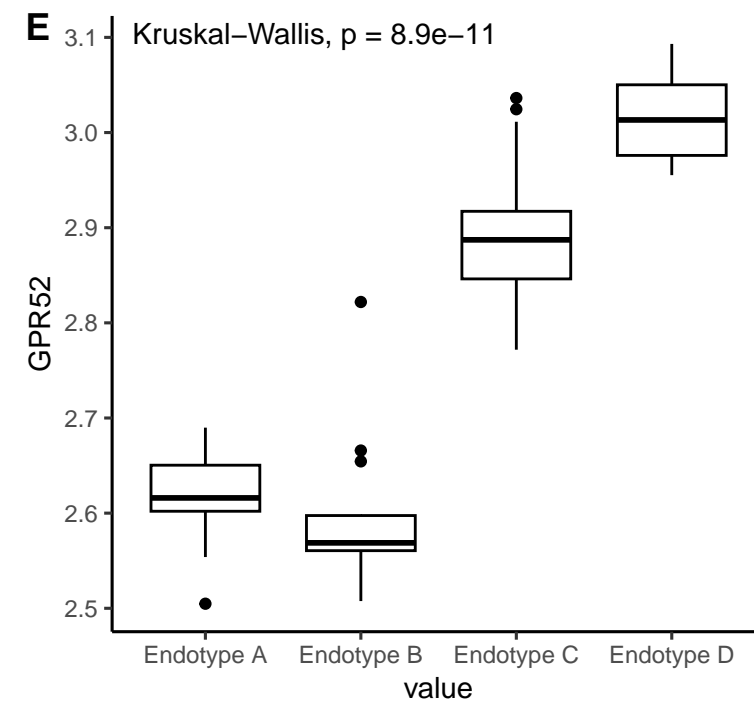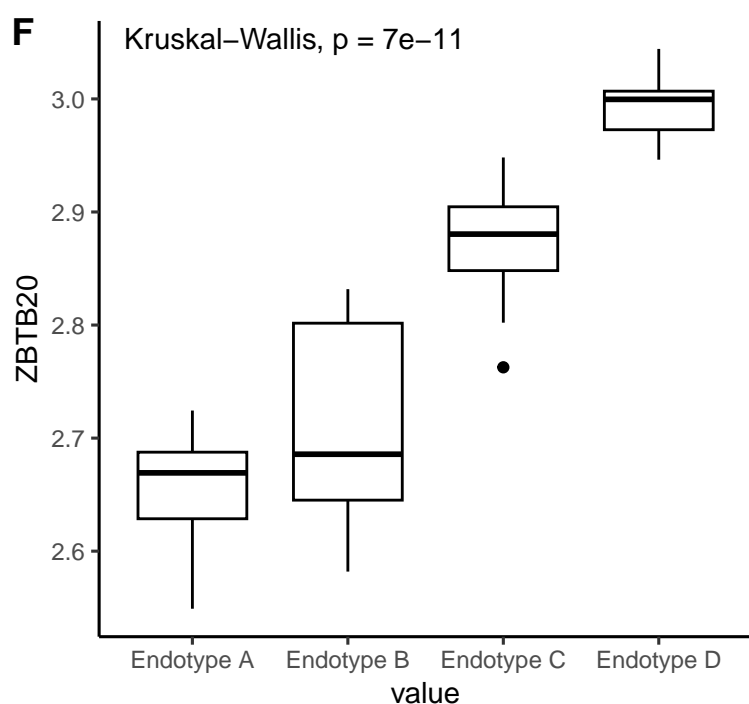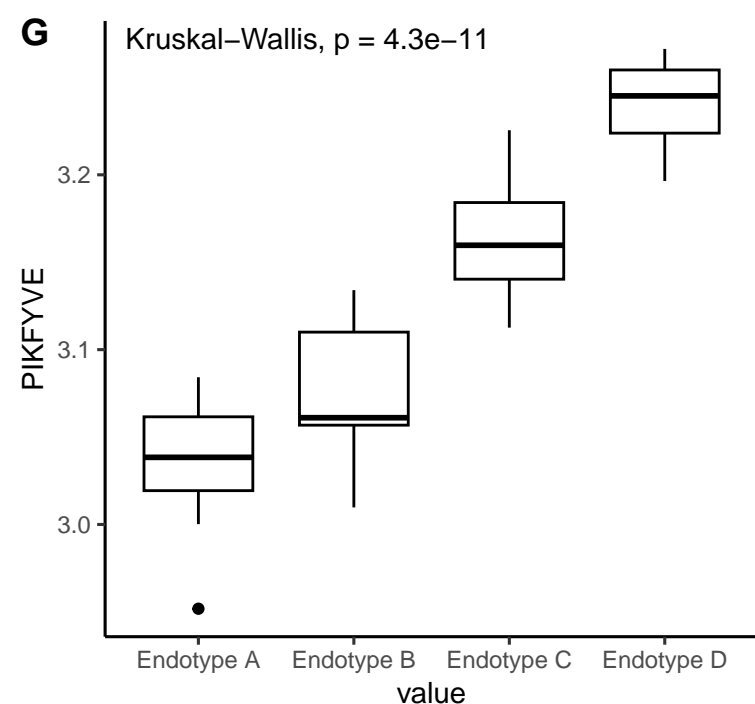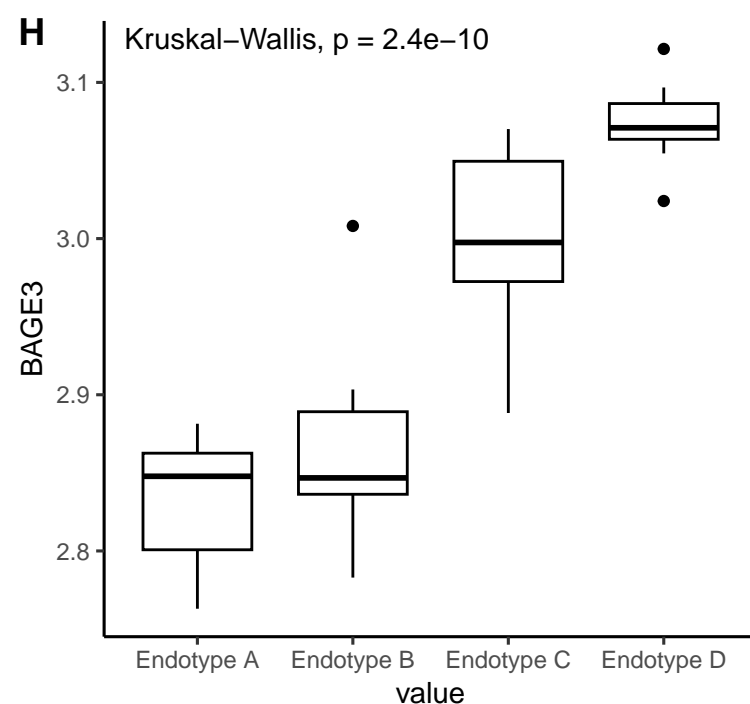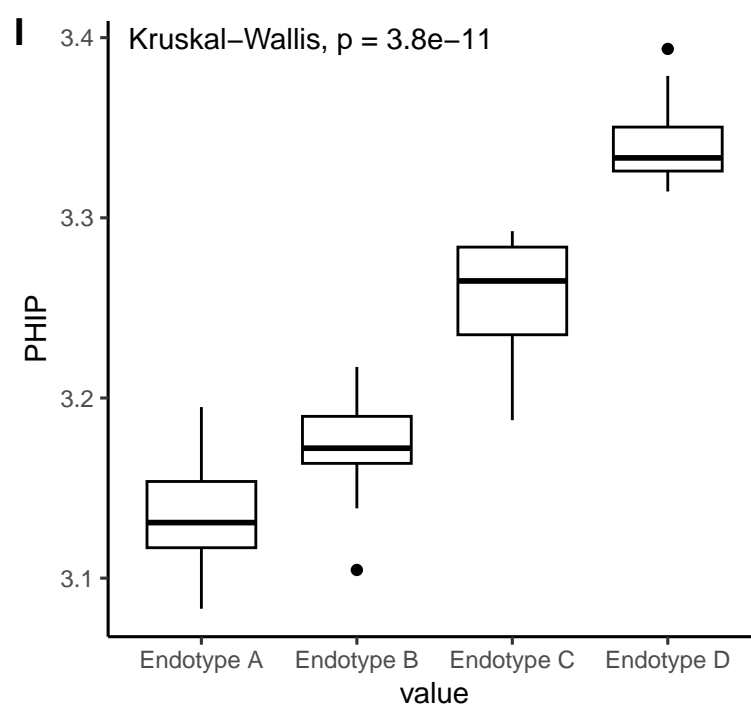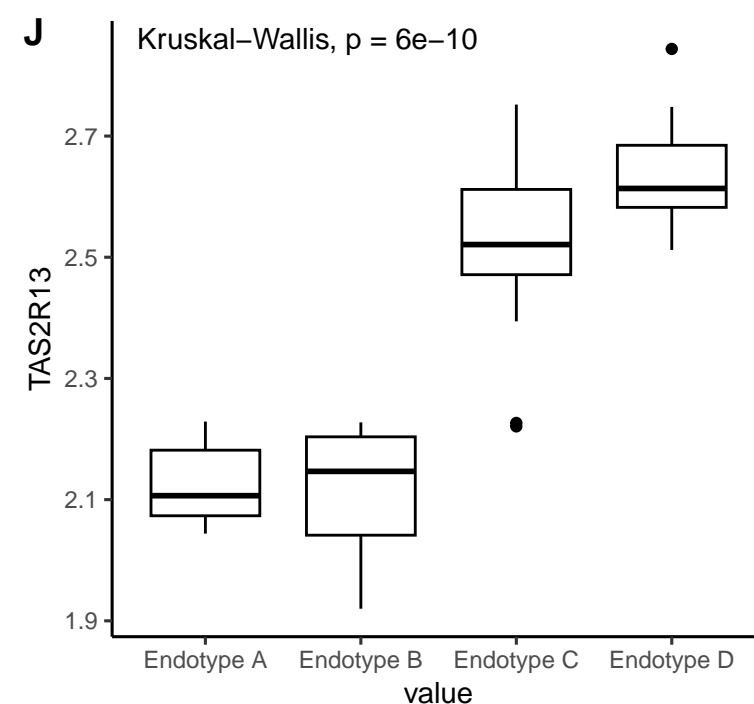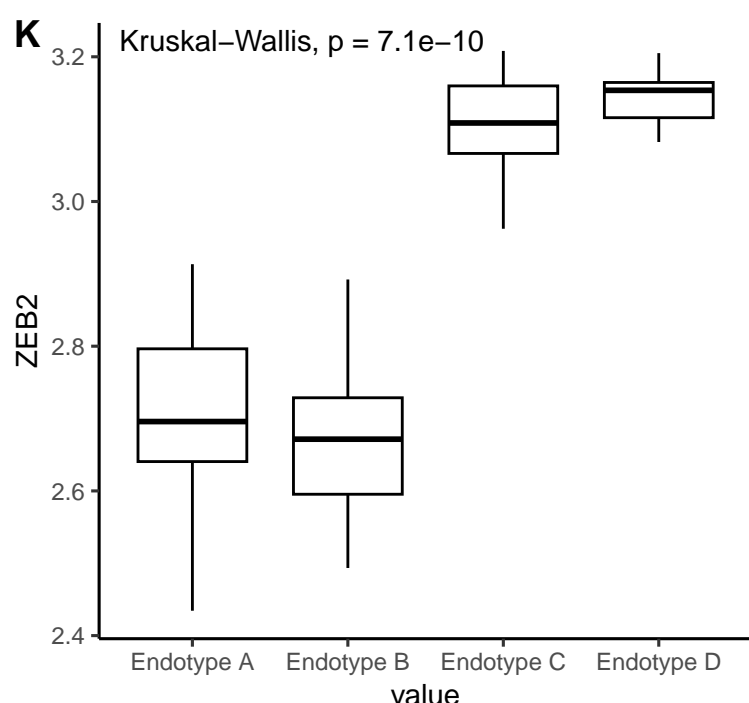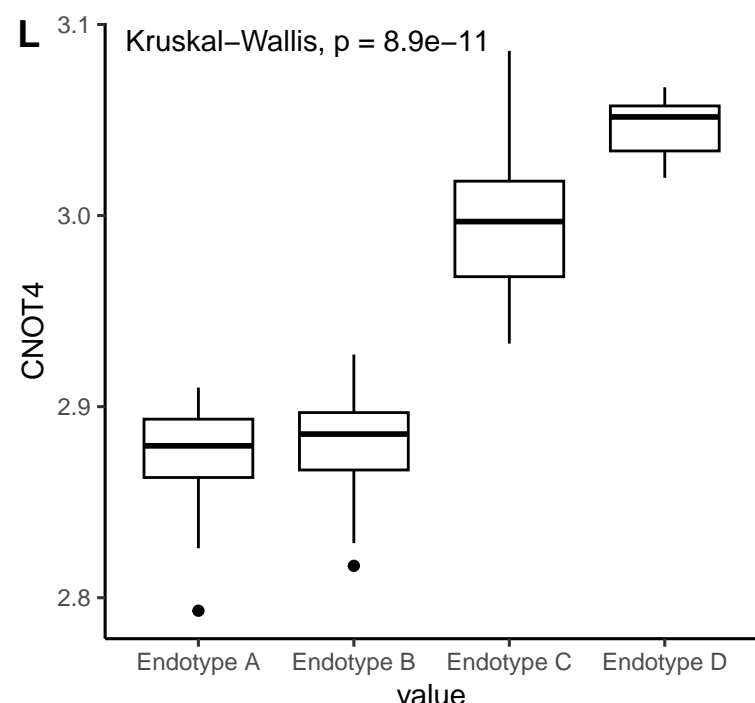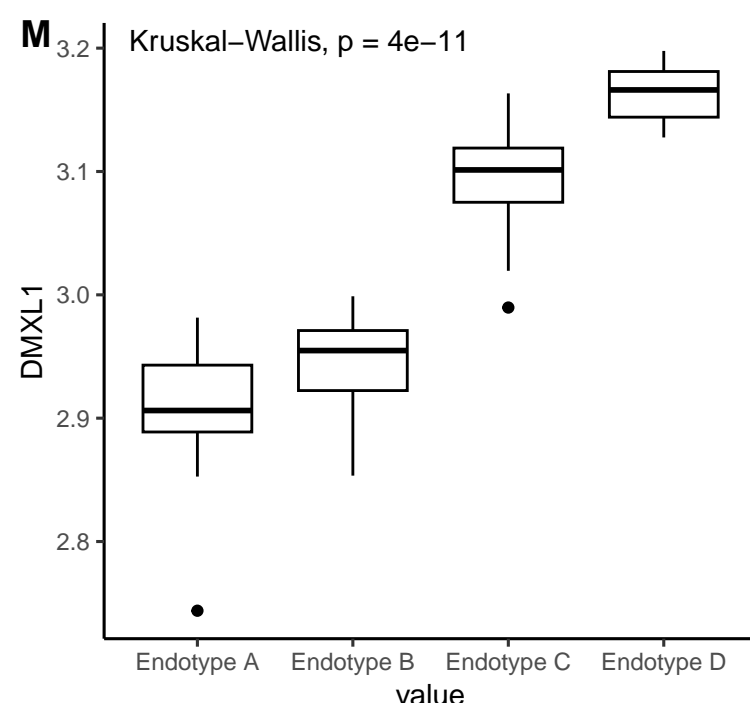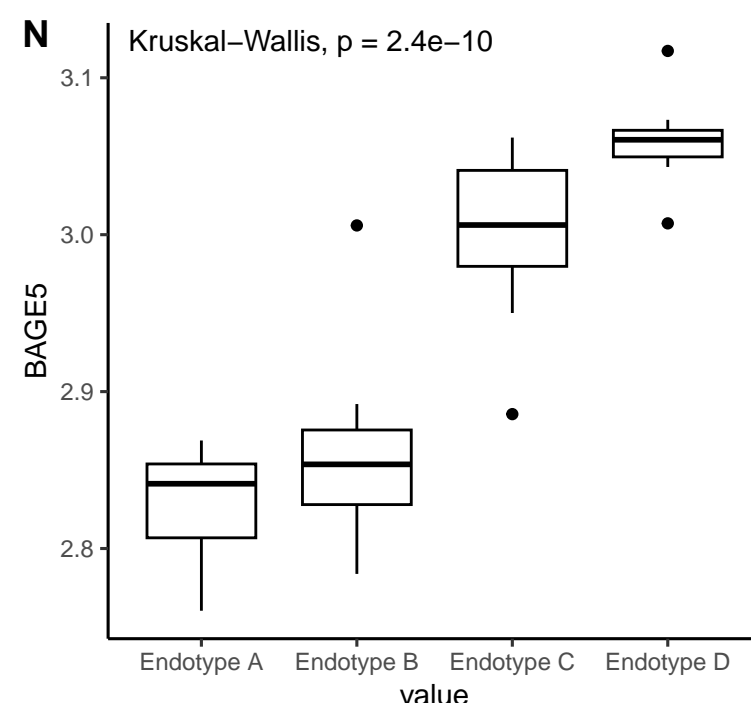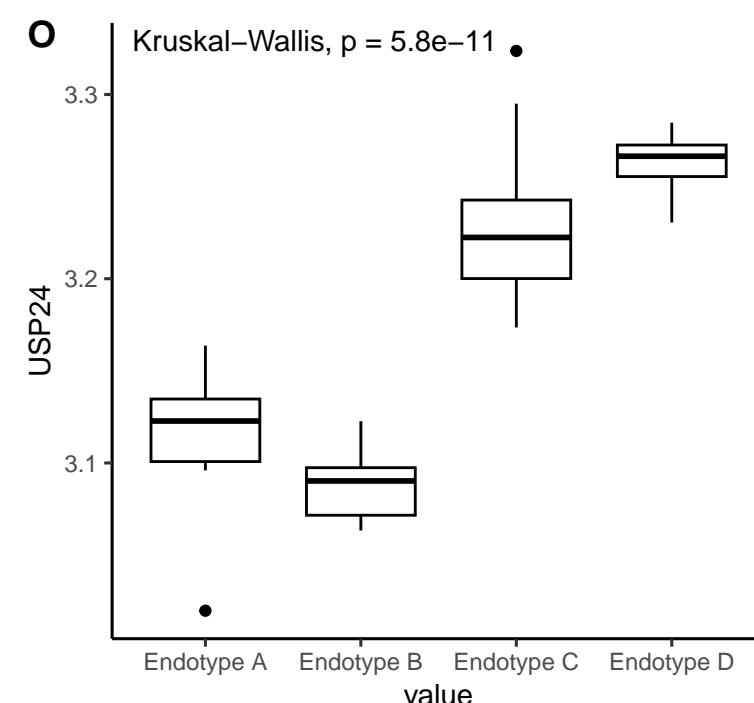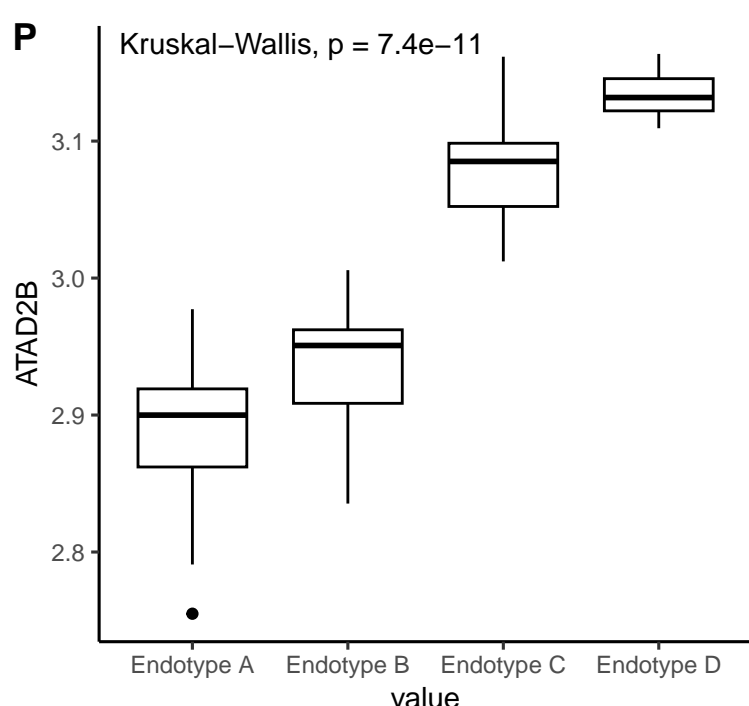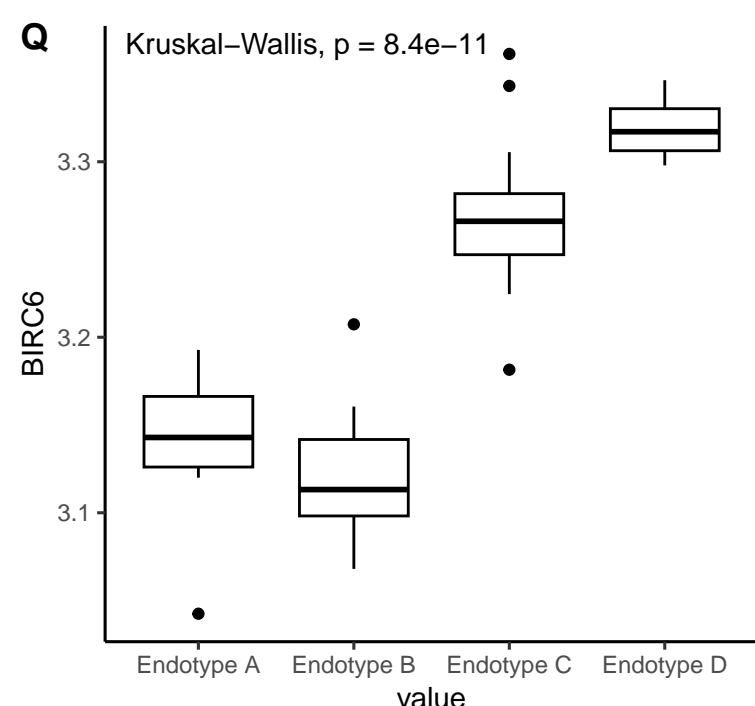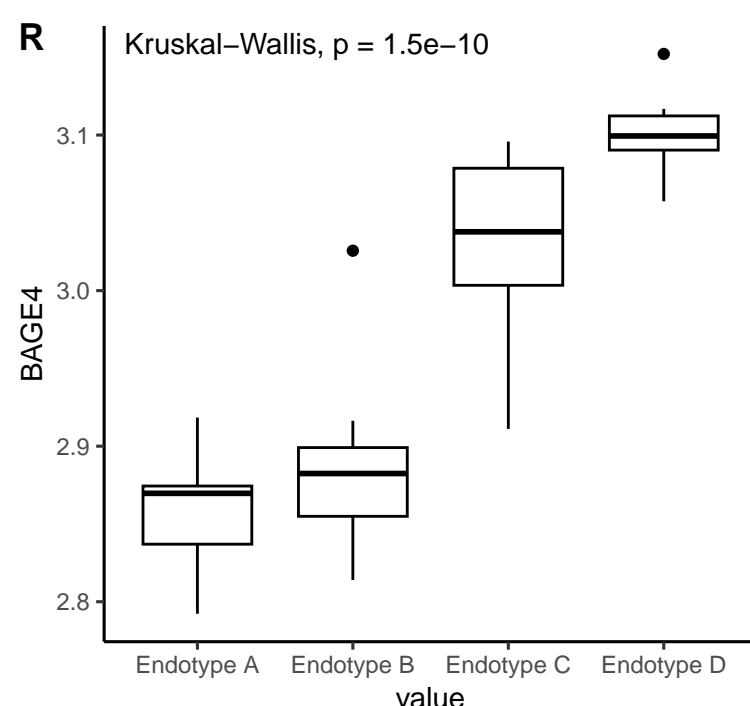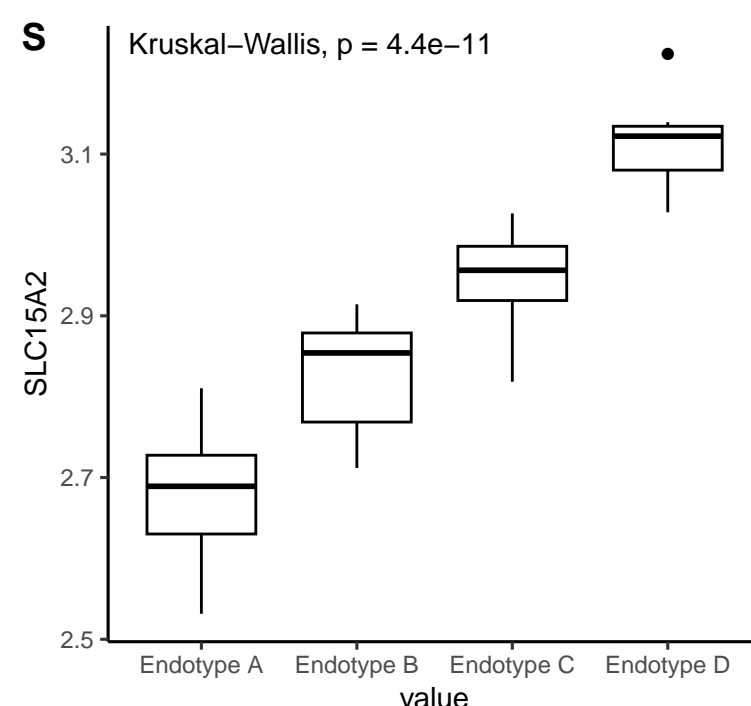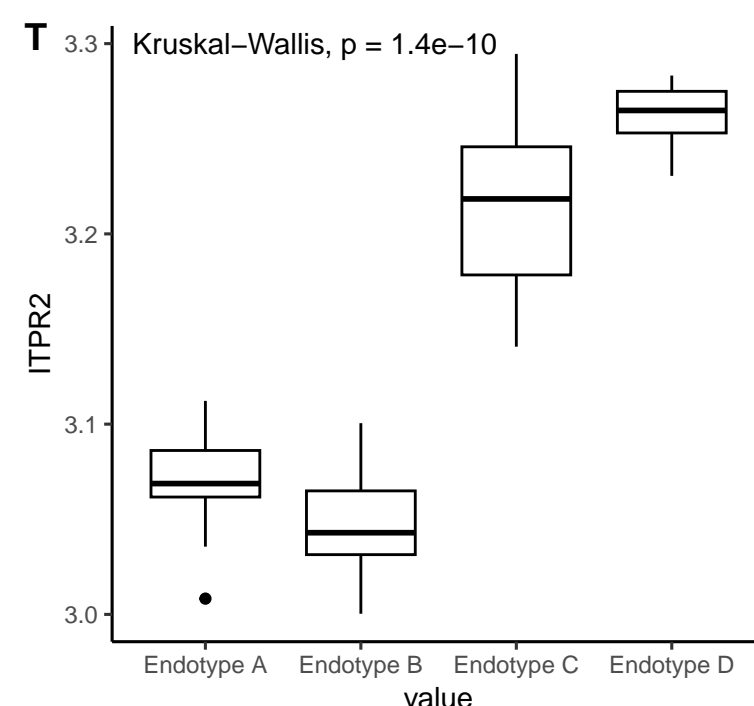

Supplement: Supplementary file 5 — Additional file 5: Supplementary Fig. 5. Boxplots with median and interquartile range of expression of top 20 genes identified via machine learning that discriminates the BPD endotypes. Kruskal-Wallis test used to assess statistical differences among groups [file 12931_2023_2596_MOESM5_ESM.pdf]
